# Frailty in Stroke—A Narrated Review

**DOI:** 10.3390/life11090891

**Published:** 2021-08-28

**Authors:** Ebrahim Bani Hassan, Steven Phu, Elyce Warburton, Nihara Humaith, Tissa Wijeratne

**Affiliations:** 1 Department of Medicine, Australia Institute of Muscular Skeletal Health, Western Health, The University of Melbourne, 176 Furlong Road, St Albans, VIC 3021, Australia; ebrahim.bani@unimelb.edu.au (E.B.H.); steven.phu@unimelb.edu.au (S.P.); 2 Falls, Balance and Injury Research Centre, Neuroscience Research Australia (NeuRA), Randwick, NSW 2031, Australia; 3 Department of Neurology, Sunshine Hospital, 176 Furlong Road, St Albans, VIC 3021, Australia; Elyce.Warburton@wh.org.au (E.W.); niharahumaith@yahoo.com (N.H.); 4 Department of Public Health, La Trobe University, Bundoora, VIC 3083, Australia; 5 Department of Medicine, Faculty of Medicine, University of Rajarata, Saliyapura, Anurdhapura 50008, Sri Lanka

**Keywords:** stroke, frailty, serial systemic immune-inflammatory indices (SSIIi)

## Abstract

This narrative review provides a summary introduction to the relationship between stroke and physical and cognitive frailty syndromes and the neuro-inflammatory similarities (including inflammaging) between the two. The review argues the potential effects of Post COVID-19 Neurological Syndrome (PCNS, also known as Long COVID) with similar pathophysiology. Many patients who have suffered from acute stroke experience long-lasting symptoms affecting several organs including fatigue, brain fog, reduced physical activity, loss of energy, and loss of cognitive reserve, culminating in the loss of independence and poor quality of life. This is very similar to the emerging reports of PCNS from different parts of the world. Stroke, particularly in older adults with comorbidities appears to impact the health and welfare of patients by reducing central neuronal input and neuromuscular function, with muscular atrophy and neuropsychiatric complications. The cumulative effects can potentially lead to a range of physical and cognitive frailty syndromes, which, in many cases may be attributed to persistent, maladapted, low grade, chronic inflammation. Meanwhile, post-COVID-19 Neurological Syndrome (also known as Long COVID Syndrome) appears to share a similar trajectory, adding further urgency for investigations into the mechanisms underlying this constellation of symptoms.

## 1. Introduction

### 1.1. Stroke

Stroke is a leading cause of morbidity, disability, dependency and mortality globally. Age-adjusted stroke incidence ranges from 76 to 119/100,000/year depending on the country [1,2]. Taking the global average (97.5/100,000/year) and a world population of 7.6 billion people we can estimate that 7.41 million new cases occur yearly, translating to one case every 4.3 s, a number which is ever-increasing [3]. Post-stroke, over 60% of patients experience some form of disability, with half severely so. Of this, 50% of patients will experience persistent hemiparesis and 30% will remain permanently unable to walk or undertake other activities of daily living without assistance [4]. Furthermore, given the declining average age of stroke occurrence [5] and findings that one-quarter of stroke survivors are of working age in categories of work defined as a “major and experienced” [5], the resultant impacts on daily society are significant.

Due to the brain’s physical and functional connection to all bodily systems, adverse events resulting from catastrophic brain injuries such as stroke are manifested in a wide range of syndromes from immunodeficiency [6] to musculoskeletal dysfunction [7,8,9], concurrent cardiovascular disease [10], central post-stroke pain [11], neurogenic bladder [12] and cognitive frailty [13]. This diverse range of outcomes may occur regardless of the location of damage, particularly if the part of the brain directly controlling the organ/system is intact [14]. Moreover, psychiatric complications from stroke are well documented and include depression, functional disability, fatigue, cognitive impairment and decreased sexual activity [15,16,17,18,19]. Musculoskeletal complications of stroke include spasticity, osteoporosis and sarcopenia, deformities of joints and consequent fractures, which often contribute to the transition towards frailty. Indeed, pre-frailty and frailty have been linked to a high risk of cardiovascular disease and stroke [10].

### 1.2. Frailty

Despite the lack of a consensus definition for frailty, recurrent themes in the description of frailty as a syndrome include elements of reduced physiological reserve, vulnerability, a typical phenotype, characteristics, pathogenesis and adverse outcomes [20]. Summarised, it encompasses a state of vulnerability in which the individual’s ability to cope with stressors is diminished, placing them at increased risk for adverse health outcomes [21]. In community-dwelling older adults, frailty is present in 9.9% of older adults, with women at greater risk compared to men [22].

### 1.3. Diagnosis of Frailty

Whilst the concept of frailty and a general understanding of how the frail patient presents can be simply described, the process of diagnosing frailty has only recently been formalised with assessment tools. There remains a lack of consensus regarding the best method of assessment of frailty, with two commonly used definitions being the Frailty Phenotype (FP) [23] described by Fried and the Frailty Index (FI) proposed by Rockwood [24].

The FP considers frailty as a physical model comprised of five criteria: reduced energy levels (exhaustion), low muscle strength (weakness), weight loss, slow gait speed and low physical activity levels [23]. Individuals are given a score according to the number of criteria they fulfil, and thus are classified as being fit (no criteria evident), pre-frail (1 to 2), or frail (≥3). 

In contrast, the FI takes a broader view of frailty as an accumulation of deficits, using a 60-point scale that considers social and psychological factors in addition to clinical observations [24]. Compared to the FP, the FI requires a comprehensive assessment of the individual’s circumstances, viewing frailty as a holistic rather than a single insult. 

The FI is considered more specific and accurate than the FP because it is finely graded [25]. Whilst the FI has greater value when investigating the pathogenesis and underlying mechanism of frailty, the inclusion of more deficits associated with adverse health outcomes, may also affect its feasibility and efficacy as a diagnostic tool. Furthermore, the FI, directly and indirectly, includes measures of both disability and comorbidity by including their associated deficits. Meanwhile, the FP limitations lay in the fact that one cannot understand the aetiology and mechanisms of frailty through it, because calculating the number of accumulated deficits does not establish a syndrome in a clinical setting [26]. Furthermore, the FP, whilst being easily assessed in clinical practice, utilises a scale whereby small changes result in significant progression in classification.

### 1.4. Aetiology of Frailty as a Musculoskeletal Disorder 

The aetiology of frailty is multifactorial, involving a complex interplay of multiple organ systems and biological mechanisms that results in an increasing ‘allostatic load’ [20]. The health of the musculoskeletal system—in particular muscular strength and gait speed—plays a significant role in defining not only frailty but also sarcopenia (which is defined as the loss of both muscle mass and strength) [27]. Reciprocally, recent studies have shown the positive impact of exercise as a preventive intervention in both sarcopenia and frailty [28].

Chronic inflammation, specifically elevated levels of IL-6, C-reactive protein and tumour necrosis factor-α, has been associated with frailty [29]. Low-grade inflammation has negative effects on the musculoskeletal and endocrine systems, particularly over time, with the term “inflammaging” coined to describe this process of deterioration [30].

Age-related endocrine system changes (reductions in sex steroids [oestrogen and testosterone] and Insulin-like Growth Factor-1) have also been found to have negative effects on muscle mass and strength [31]. The impacts of hormonal changes due to ageing are wide-ranging, increasing the risk of cardiovascular, metabolic and musculoskeletal diseases. 

Given the high rate of frailty already present in healthy community-dwelling older adults, it is evident that the experience of stroke will further exacerbate the elevated risk. Considering the common risk factors that predict the severity of both conditions, this narrative review will discuss the potential causes of disability and frailty in stroke patients.

## 2. Frailty in Stroke Patients

The aetiologies of frailty in vascular brain insults are multifactorial and can be categorised into the following three groups:

### 2.1. Decreased Load-Bearing

The musculoskeletal system requires axial loading to prevent it from atrophying [32]. A rapid decline in muscle volume shortly after stroke has been documented, secondary to stroke-related catabolic overactivation and anabolic blunting [8]. This is particularly prominent on the side of the body contralateral to the lesion, but also—though to a lesser degree—affects the ipsilateral side [8,33]. Nearly 60% of individuals post-stroke do not meet the recommended activity guidelines [34]. This both results from and leads to a further decline in load-bearing, with potential for ongoing muscular atrophy and decreasing bone mineral density over time. 

Immobilisation is the primary cause of increased bone resorption and losses in bone mineral density (BMD), which peak at the first year post-stroke, whilst the degree of residual weakness and vitamin D levels are particularly relevant to longer-term changes in BMD [9,35]. Given the importance of weight-bearing and physical activity, it is not surprising to find those who are unable to overcome the resistance of gravity in the manual muscle test also present with low BMD in the neck of the femur. Furthermore, disability reported via the Modified Barthel Index was correlated with decreased BMD in the femoral neck [36]. 

In keeping with the induced generalised sarcopenia in animal models, strength in both the affected and non-affected limbs is reduced post-stroke, with significant differences found between stroke and control subjects [7,8]. Furthermore, strength deficits in the lower extremities post-stroke play a significant role in decreasing participation in daily and social activities [37], possibly contributing to a cycle of declining activity, losses in muscle strength, and ultimately the development of frailty.

### 2.2. Motor-Cognitive Pathways Decline 

Motor-cognitive profiles have been defined in chronic neurodegenerative conditions such as Alzheimer’s disease/mild cognitive impairment, frontotemporal degeneration, vascular cognitive impairment, amyotrophic lateral sclerosis, and Parkinson’s disease [38]. Cerebral small vessel diseases, particularly of the white matter, are also associated with frailty [39]. There are no reports, however, on whether nervous deficits (e.g., impaired proprioception), cognitive decline, or muscle mass decline (due to inactivity and denervation) contribute to frailty in stroke patients. 

Recent reports on Post COVID-19 Neurological Syndrome (PCNS) raise low-grade persistent inflammation as a catalyst of fatigue and brain fog, thereby contributing to the development of frailty following neurological infections [40,41]. This shared pathobiology of COVID-19 and stroke is well characterised [42]. Understanding and mitigating the disease mechanisms of both these conditions in relation to frailty is of the greatest importance in promoting better long-term health.

Cognitive frailty can be defined as a “heterogeneous clinical manifestation characterised by the simultaneous presence of both physical frailty and cognitive impairment” [43]. It is possibly a combination of muscular, proprioceptive and cognitive factors (centrally driven through persistent low-grade inflammation), but the proportional role of each factor or other possible causal variables is yet to be determined in the various conditions where cognitive frailty is common (including stroke, or systemic insults such as COVID-19). Interestingly, it has recently been shown that the risk of dementia grows by seven times for patients whose gait velocity declines, by over three times in those with cognitive decline and by eight times in those with combined gait velocity and cognitive declines [44]. Hence, motor decline should be dealt with as a clinical risk factor for both physical and cognitive decline and further exploration of motor-cognitive pathways is mandated.

### 2.3. Inflammatory Mediators and Other Potential Factors

The serum levels of post-stroke inflammatory markers in predicting future stroke risk [42,45,46,47], cognitive impairment [48], musculoskeletal involvement [7,8,9,35], depression [16,19], prognosis or long-term disability outcome [15,49] are being studied widely. Studies that have evaluated cognitive impairment in stroke patients have found associations with a range of common inflammatory markers including interferons 1, 2, 6, 8, 10, 12 and gamma, C-reactive protein, and tumour necrosis factor [50,51,52,53]. These markers are also associated with muscle and bone volume decline. 

Other less commonly investigated inflammatory markers considered as risk predictors following lacunar stroke include [45]: high sensitive C reactive protein (HsCRP), serum amyloid A (SAA), soluble CD40 Ligand (CD40-L), tumour necrosis factor receptor-1 (TNFR1) and monocyte chemoattractant protein-1 (MCP-1). Meanwhile, inflammatory markers such as alpha-2macroglobulin (A2M), baseline serum amyloid protein (SAP) and pre-post tissue-plasminogen activator (tPA) variations (Δ) of metalloproteinase 9 are considered as markers of poor outcomes [54].

Wijeratne and Wijeratne showed the clinical utility of serial systemic immune-inflammatory indices (SSIIi) in the case of PCNS with the help of easily available, universal serial white cell counts in the context of COVID-19 and PCNS (Long COVID). This can be explored in the field of stroke and frailty to elucidate the relationship between low-grade inflammation and cognitive frailty.

As demonstrated above, ischaemic and/or inflammatory injuries to the brain can lead to frailty through multiple mechanisms, directly or indirectly. However, the associations between inflammatory markers and frailty, cognitive decline and musculoskeletal atrophy are part of a bigger picture of the brain and body connection. Routine use of full serum protein profile studies and more easily available biomarkers such as SSIIi in diseases like stroke may help determine stronger markers or causal factors of such conditions to enable more targeted investigations and therapies. 

## 3. Conclusions 

Stroke often results in an extensive and irreversible reduction in function, independence, and quality of life. In many cases, there is a strong overlap between the long-term effects of stroke and the development of frailty resulting from interactions between multiple factors including the musculoskeletal system, immune system, and cognitive pathways. Understanding the immunoinflammatory mechanisms and biomarkers of frailty could provide new insights into the diagnosis and prevention of disability and frailty in stroke patients. The recent observation on shared pathobiology between stroke and systemic inflammatory conditions like COVID-19 has created an incentive and opportunity to advance research in this direction. Achieving optimal outcomes for stroke patients lies in the early diagnosis and prevention of frailty and its associated decrease in both quality and quantity of life.

## Data Availability

Not applicable.
